# Successful Ablation for Atrial Tachycardia Originated from Sinus Venosa with Tachycardia-Induced Cardiomyopathy

**DOI:** 10.1155/2016/4865034

**Published:** 2016-10-20

**Authors:** Sou Takenaka, Hideaki Sato, Mikio Yuhara, Takashi Uchiyama

**Affiliations:** Department of Cardiovascular Medicine, Toda Chuo General Hospital, Toda, Japan

## Abstract

A 74-year-old male suffering from congestive heart failure with atrial tachycardia (AT) with 2 : 1 atrioventricular conduction was admitted to our hospital. After the therapy with diuretics and *β*-blocker, his rapid AT was still sustained. He took the catheter ablation for his AT. Postpacing interval mapping from entrainment and noncontact mapping system revealed the mechanism of his AT, originated from sinus venosa. His AT was successfully terminated and eliminated by radiofrequency catheter ablation. After the successful ablation, he has been free from any AT, and his cardiac function was also improved.

## 1. Introduction

Tachycardia-induced cardiomyopathy is considered reversible once the tachyarrhythmia is controlled with medication or ablation. Atrial tachycardia (AT) can be causing tachycardia-induced cardiomyopathy [1]. Entrainment mapping and electroanatomical mapping can reveal the mechanism of AT. However, in some cases of AT, it is sometimes difficult to identify the mechanism.

AT is rarely originated from sinus venosa (SV) [2]. A functional line of conduction block is often observed in SV [3, 4]. We report a case of tachycardia-induced cardiomyopathy in a patient who successfully underwent catheter ablation for AT originated from SV.

## 2. Case Presentation

**Figure 1:**
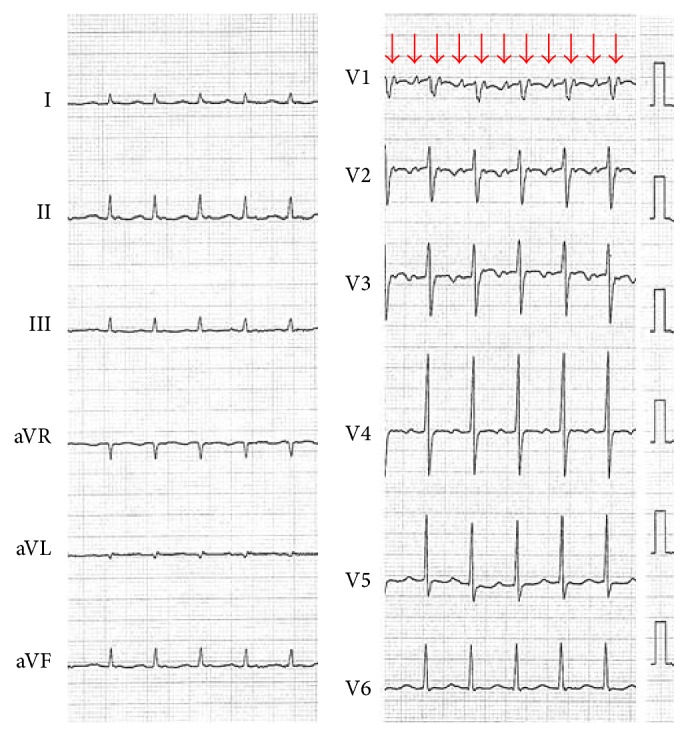
Electrocardiogram revealed atrial tachycardia (AT) with 2 : 1 atrioventricular conduction. P wave (red arrow) morphology was positive in inferior leads (II, III, and aVF), positive in lead V1, and isoelectric in aVL.

A 74-year-old male suffering from shortness of breath for 2 months was admitted to our hospital. His chest X-ray revealed expansion of CTR and pleural effusion. His electrocardiogram showed atrial tachycardia (AT) with 2 : 1 atrioventricular conduction (Figure 1). P wave morphology was positive in inferior leads (II, III, and aVF) and positive in lead V1. Ultrasound cardiography revealed a poor left ventricular ejection fraction (LVEF = 22%). His AT resulted in cardiac failure with tachycardia-induced cardiomyopathy. After the treatment for the heart failure with anticoagulant therapy, he underwent an electrophysiological study.

**Figure 2:**
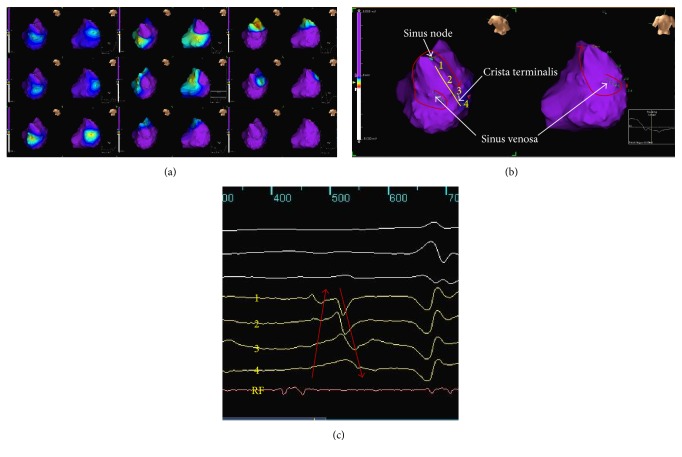
(a) Activation mapping in right atrium revealed that the earliest activation site was sinus venosa. (b) The activation site was moved to the high right atrium and down along the crista terminalis. (c) Virtual unipolar electrogram on the crista terminalis. Red arrow = traces of the movement of activation site; yellow line = crista terminalis; RF = ablation catheter (crista terminalis).

**Figure 3:**
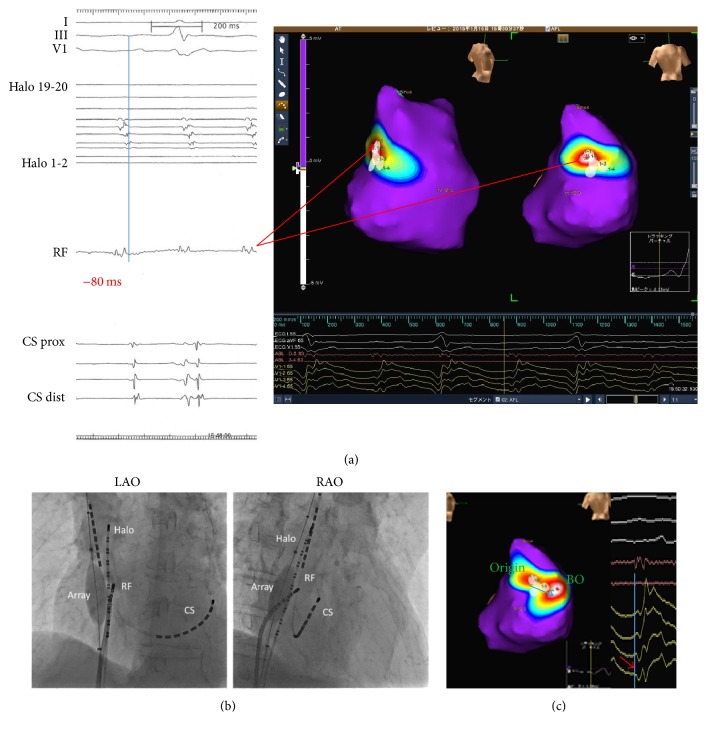
(a) Double potentials were recorded and preceded the onset of P wave by 80 ms. (b) Catheter position at RF site. RF catheter was located at posterior medial wall. RF: ablation catheter; CS: coronary sinus; prox: proximal; dist: distal. (c) The noncontact unipolar electrogram revealed a QS pattern of the origin (red allow). The length of preferential pathway (blue arrow) was 8 mm. BO = breakout site of AT.

His tachycardia cycle length (TCL) was 230 ms, and the postpacing intervals (PPIs) from entrainment of cavotricuspid isthmus (280 ms), the lateral site of tricuspid annulus (260 ms), and the ostium of coronary sinus (340 ms) were long PPI-TCL (>30 ms). The PPI from the posterior wall of high right atrium (240 ms) matched his TCL closely. We inserted and positioned the multielectrode array into the right atrium. The dynamic activation map during his AT revealed focal AT arising from the posterior medial wall of high right atrium. The activation site was moved to the high right atrium and down along the crista terminalis (Figures 2(a), 2(b), and 2(c)). The earliest potential preceded the onset of the P wave at the surface ECG by 80 ms (Figure 3(a)). In addition, the local electrogram at the site of tachycardia origin recorded the double potential (Figure 3(a)). The noncontact unipolar electrogram revealed a QS pattern of the origin (Figure 3(b)). In addition, 8 mm of preferential pathway existed (Figure 3(c)). We defined that his AT was originated from the SV. Radiofrequency (RF) energy delivery at the site of this origin slowed the tachycardia cycle length and finally resulted in sinus rhythm within 15 seconds. There was no inducible tachycardia thereafter, and his cardiac function was also improved (LVEF = 50%).

## 3. Discussion

The SV is located at the posteromedial right atrium. In this case, during atrial flutter, a functional blockline is seen at the SV [5]. Park et al. reported that focal AT which develops during atrial fibrillation ablation is rarely originated from SV [2]. In this case, the earliest activation site was the posterolateral right atrium, and double potentials were recorded at this site. We defined that his AT was arising from SV and successfully terminated using focal RF application at that site.

The algorithm of the P wave is helpful for prediction of AT foci. In this case, P wave morphology during AT wasisoelectric in inferior leads (II, III, and aVF), positive in V1 lead, and isoelectric in aVL. Kistler et al. [6] reported that a negative or positive-negative biphasic P wave in lead V1 was associated with specificity of 100% for right atrial tachycardia, and positive or negative-positive biphasic in lead V1 was associated with that of 100% for left atrial tachycardia. Park et al. [2] reported that negative or positive-negative biphasic P wave was shown in only 70% of patients with AT arising from sinus venosa.

To our knowledge, SV has not previously been reported as the focal origin of AT [7]. SV is located in the posterior medial wall of the RA rather than posterior lateral wall. It also has a functional blockline [5] and low-voltage area [3], which was critical for the reentrant circuit in right atrium. In this case, success site of RF catheter was positioned in the posteromedial wall of the RA (Figures 2(a), 2(b), and 3(b)). In addition, the right atrial activation mapping on noncontact mapping system suspected that the earliest activation site was SV. The local electrogram at this site recorded the double potential. The diagnosis was confirmed by entrainment pacing showing the shortest postpacing interval at SV. Focal RF ablation at SV was successful in eliminating AT. The mechanism of his AT was focal and microreentrant [8]. The preferential pathway was present, and its length was about 8 mm. This finding suggested that his SV might have some remodeling, such as fibrosis [8].
